# The state of Sergipe contribution to GH research: from Souza Leite to Itabaianinha syndrome

**DOI:** 10.20945/2359-3997000000567

**Published:** 2022-11-28

**Authors:** Manuel H. Aguiar-Oliveira, Roberto Salvatori

**Affiliations:** 1 Universidade Federal de Sergipe Programa de Pós-graduação em Ciências da Saúde Divisão de Endocrinologia Aracaju SE Brasil Divisão de Endocrinologia, Programa de Pós-graduação em Ciências da Saúde, Universidade Federal de Sergipe, Aracaju, SE, Brasil; 2 The Johns Hopkins University School of Medicine Baltimore Department of Medicine Division of Endocrinology, Diabetes and Metabolism Maryland USA Division of Endocrinology, Diabetes and Metabolism, Department of Medicine, The Johns Hopkins University School of Medicine Baltimore, Maryland, USA

**Keywords:** GH, GHRH receptor, IGF1, acromegaly, growth hormone deficiency

## Abstract

In the late 19^th^ century, José Dantas de Souza Leite, a physician born in Sergipe, published the first detailed clinical description of acromegaly under the guidance of the French neurologist Pierre Marie. In 2014, the Brazilian Society of Endocrinology and Metabolism created the “José Dantas de Souza Leite Award”, which is granted every two years to a Brazilian researcher who has contributed to the development of endocrinology. In 2022, the award was given to another physician from Sergipe, Manuel Hermínio de Aguiar Oliveira, from the Federal University of Sergipe for the description of “Itabaianinha syndrome” in a cohort of individuals with isolated GH deficiency due to a homozygous inactivating mutation in the GH-releasing hormone receptor gene. This research, which was carried out over almost 30 years, was performed in partnership with Roberto Salvatori from Johns Hopkins University and in collaboration with other researchers around the world. This review article tells the story of Souza Leite, some milestones in the history of GH, and summarizes the description of Itabaianinha syndrome.

## PRELIMINARY CONCEPTS

Before reporting the history from Sousa Leite on Itabaianinha syndrome, we need to clarify concepts that are used in the text. While the ability to grow is a characteristic of all living beings, growth hormone (GH) is an achievement of vertebrates to increase their body, increasing their ability to reproduce and to obtain food. Therefore, we call the “somatotrophic system” all the mechanisms involved in growth, that is, the somatotrophic axis and the extrapituitary circuits. The first factor, which is critical for body size, includes the hypothalamic factors GH-releasing hormone (GHRH), somatostatin, ghrelin, pituitary GH, and circulating (or “endocrine”) insulin-like growth factor 1 (IGF1). The other circuit, the extrapituitary circuits, which is relevant for body functions, comprises insulin, IGF2, and the local production of GH, IGF1, IGF2, IGF binding proteins (IGBPs) and several growth factors, such as fibroblast growth factor (FGF), vascular endothelial growth factor (VEGF), platelet-derived growth factors (PDGFs), transforming growth factor-β (TGF-β), and connective tissue growth factor (CTGF), acting in different tissues. In this article, we will highlight the roles of the components of the somatotrophic system by studying congenital and severe isolated GH deficiency (IGHD). In this article, we highlight the roles of the components of the somatotrophic system in relation to the findings of our studies performed on a cohort of Itabaianinha subjects presenting with congenital, severe, isolated GH deficiency (IGHD) due to a homozygous inactivating mutation in the GH-releasing hormone receptor gene.

## JOSÉ DANTAS DE SOUSA LEITE

**Figure 1:**
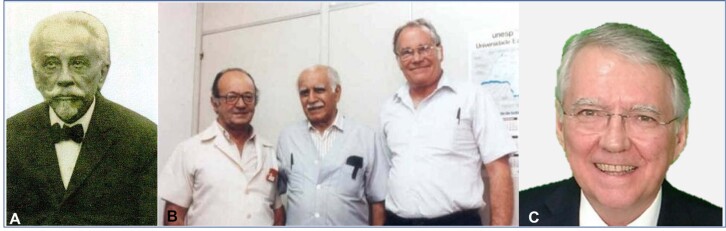
**A.** José Dantas de Souza Leite (1859-1925). Physician born in Sergipe, one of the pioneers in the description of acromegaly, under the guidance of the famous French neurologist Pierre Marie.(Source: public domain photo from http://anm.org.br accessed July 5, 2020). **B**. From the reader's left to the right, José Antunes -Rodrigues (1933-), Miguel Covian (1913-1992) and Samuel McCann (1925-2006) working together at USP's Ribeirão Preto School of Medicine.(Source: private collection Ayrton C. Moreira). **C**. Ayrton Custódio Moreira (1949-), Full Professor at the Department of Internal Medicine at USP's Ribeirão Preto School of Medicine and mentor of the first author of this manuscript, Manuel H. Aguiar-Oliveira.

Source: private collection Ayrton C. Moreira)

Sergipe is the smallest Brazilian state located in the northeast of the country. Perhaps due to its small size, its endocrinological vocation is linked to the study of GH disorders (1). One of the pioneers of studies on acromegaly, the physician José Dantas de Souza Leite (1859-1925) (Figure 1A) was born in the southwest of Sergipe in the city of Santa Luzia do Itanhy. He graduated in Medicine at the first Faculty of Medicine in Brazil (*Faculdade de Medicina da Bahia*), which was created in 1808 by Prince João VI after the transfer of the Portuguese throne to Brazil, when Portugal was about to be invaded by Napoleon Bonaparte's troops. Afterwards, Souza Leite graduated again in Medicine in Paris, where he attended the service of Prof. Charcot at the Salpêtrière Hospital and became a disciple of the renowned neurologist Pierre Marie (2,3). In 1886, Pierre Marie coined the term “acromegaly” to describe a deforming condition associated with the growth of extremities, which was already described in the 16th century by the Dutch physician Johannes Wier but poorly understood until then (4). Four years later, Souza Leite presented his doctoral thesis “De l'acromégalie: maladie de Marie”. He thoroughly described the clinical picture, evolution, differential diagnosis, prognosis, treatment, and pathology of the pituitary gland in acromegaly. In 1891, the New Sydenham Society of London published the book “Essays on Acromegaly”, which was authored by Pierre Marie and Souza Leite and translated into English (5). Souza Leite was internationally acknowledged for his contribution to the initial characterization of acromegaly one century after his death occurred at age 66 in Rio de Janeiro as a full Member of the National Academy of Medicine (3). In 2014, the Brazilian Society of Endocrinology and Metabolism (Sociedade Brasileira de Endocrinologia e Metabologia/SBEM) created the “José Dantas de Souza Leite Award”, which is granted every two years to a Brazilian researcher who has contributed to the development of Endocrinology (3), and its first winners included Licio Augusto Velloso (Campinas University, São Paulo), Berenice Bilharinho de Mendonça (São Paulo University, São Paulo), and Ana Luiza Maia and Poli Mara Spritzer, who were both from the Federal University of Rio Grande Sul. In 2022, Manuel Hermínio de Aguiar Oliveira from the Federal University of Sergipe received this award.

## MILESTONES IN THE HISTORY OF GROWTH HORMONE

The relationship between acromegaly and the hypersecretion of GH had to wait for the demonstration of GH existence by Evans and Long in 1921 (6). Evans' contribution was crucial not only to demonstrate its existence but also to establish the first bioassays for GH. The isolation and molecular characterization of GH was accomplished by Li and Evans in 1944 (7). The concept of a circulating “sulfation factor” mediating the effects of GH in peripheral tissues was proposed in the 1950s by Salmon and Daughaday (8). Subsequently, it was proven that this sulfation factor is, in fact, a somatomedin capable of competing for insulin binding sites, implying a structural and functional homology between somatomedin and insulin (9). This led to the “somatomedin hypothesis” and further the characterization of both IGF1 and IGF2 (10).

The existence of GH releasing hormone (GHRH) was suggested in the early 1960s by Reichlin (11), who caused lesions in the hypothalamus in rats and demonstrated that the GH content in the pituitary gland decreased, suggesting the presence of a hypothalamic GHRH. GHRH was initially isolated from pancreatic tumours causing acromegaly, and later hypothalamic human GHRH was shown to be identical to the one isolated from pancreatic tumours (12–14). Subsequently, in the 1970s, Guillemin and Schally identified several hypothalamic factors, including somatostatin, for which they won the Nobel Prize in Physiology or Medicine, although Samuel McCann (1925-2006) demonstrated its existence. McCann, along with Geoffrey Harris, established the theory of hypothalamic factors (15). McCann contributed to the training of José Antunes-Rodrigues (also in Figure 1B) and Ayrton Moreira (Figure 1C) from the Faculty of Medicine of Riberão Preto at USP (FMRP/USP), who were mentors of the first author of this article. Moreira was the main inspiration behind the creation of the Endocrinology Service at the Federal University of Sergipe, where most of the following data were produced (16).

In this scientific family tree, it is also possible to demonstrate cross talk with the South American Nobel Prize winner Bernardo Alberto Houssay (1887-1971) for his discovery of the role of the anterior pituitary in the regulation of carbohydrate metabolism. Houssay trained Miguel Rolando Covian (1913-1992) (Figure 1B). Already internationally recognized, Covian accepted the invitation of Dr. Zeferino Vaz to join the faculty of the newly founded FMRP/USP as head of the Department of Physiology, bringing his prestige to this nascent institution where he worked from 1955 to 1992 (17). In his work at FMRP/USP, he successively led a group of notable researchers: José Venâncio Pereira Leite, Renato Hélios Migliorini, Carlos Renato Negreiros de Paiva, César Timo-Iaria, Andrés Negro-Vilar, Maria Carmela Lico, Anette Hoffmann, José Antunes Rodrigues, Ricardo Marseillan, Aldo Bolten Lucion, among others (18). In 1954, Zeferino Vaz invited another eminent teacher, physician, and researcher, Hélio Lourenço de Oliveira, to head the newly created Department of Internal Medicine at FMRP/USP. Hélio Lourenço brought in José Veríssimo, who introduced Ayrton Moreira to Antunes-Rodrigues. Since then, physiology and internal medicine have been studied with a fertile connection in which basic and clinical research and education with a humanistic approach have been shared (19).

Before leaving Argentina, due to problems with the Peronist government, Covian completed a postdoctoral fellowship at Johns Hopkins University in Baltimore, as the first author of this article did at the same institution under the supervision of Roberto Salvatori, the second author of this paper. Additionally, at Johns Hopkins University, Herbert Evans obtained his medical degree several years ago. Thus, people and institutions cross the paths of science, with the common objective of acquiring knowledge for the benefit of humanity.

While understanding of the roles of actors in the somatotrophic system has expanded greatly, the actual impact of GH deficiency (GHD) on the body remains controversial. Idiopathic GHD, an important cause of short stature in childhood, may disappear in adulthood, raising doubts about its nature or relevance. On the other hand, acquired GHD is often part of hypopituitarism from different aetiologies, mainly pituitary tumours, surgery, or irradiation, which are often associated with deficits in other pituitary hormones, with a lack or inadequacy of the respective replacement therapies. These circumstances make it difficult to filter the role of GHD from its muddled influences. Genetically isolated GHD (IGHD) may be an alternative to evaluate the biological impacts of GH, but it is rare, and a significant number of affected individuals receive GH replacement during childhood in the other cohorts of congenital IGHD (20).

Nearly 30 years ago, we described a large cohort of individuals with severe short stature due to congenital IGHD caused by the homozygous c.57+1G>A mutation in the GHRH receptor (GHRHR) gene (*GHRHR* OMIM n.618157), with most residing in the city Itabaianinha located just 60 km (37 miles) from Santa Luzia do Itanhy (21), Souza Leite's hometown. These subjects exhibit a classical IGHD 1B phenotype, with very low (but detectable) serum levels of GH that is accompanied, in most cases, by IGF1 concentrations close to or below the detection limit (22) and an autosomal recessive mode of inheritance.

Michael Thorner, who isolated GHRH from a pancreatic tumour causing pituitary hyperplasia and acromegaly in the 1980s, emphasized that this experiment of nature demonstrates the vital importance of GHRH in addition to its role in growth (23). Moreover, this experiment of nature is writing the natural history of IGHD through the description of Itabaianinha syndrome (20,24,25).

## ITABAIANINHA SYNDROME

As a typical IGHD 1 B case, patients with Itabaianinha syndrome showed low but not absent serum GH levels. GH peaks were lower than 1 ng/mL in both clonidine and insulin tolerance tests, and no response to GHRH was observed (26). This is combined with life-long severe reduction of circulating IGF1 and considerable IGF2 upregulation, proven by an increase in the IGF2/IGFBP-3 ratio, which is a measure of its bioavailability (22). We hypothesize that both residual GH secretion (allowing some residual GH functions and immune tolerance to exogenous GH) and IGF2 upregulation (contributing to IGF bioavailability to some vital tissues, such as the brain, eye, and teeth) may have physiological implications (20,24,25). In fact, this model of IGHD, in which most adults have never received GH replacement therapy, makes it possible to analyse the effects of the somatotrophic axis (pituitary GH and circulating IGF1) and extrapituitary circuits (IGF2 and local production of IGF1 and IGF2) on body size and body functions.

The main physical findings of the Itabaianinha untreated IGHD adult subjects were proportionate short stature, doll facies, high-pitched voice, central obesity, and wrinkled skin. However, these individuals had several additional phenotypic characteristics, arguably with a greater number of beneficial than harmful consequences to their health. They exhibited normal quality of life (27) and normal longevity (28), with increased healthspan, that is, the period of life without disabling morbidities (25). In this review, we update the consequences on body size (Table 1) and body functions (Table 2).

**Table 1:** Body size measurements in the Itabaianinha syndrome

Normal sized newborns Post-natal cumulative stature reduction Final adult stature in males : 128.7 ± 5.9 cm Final adult stature in females : 117.6 ± 5.7 cm Mean SDS: stature (−7.0), maxillary length (−6.5), total anterior facial height (−4.3) Mean SDS: head perimeter (−2.7) Mean SDS: maxillary arches (−1.6) Mean SDS: mandibular arches (−1,0) Mean SDS: mandibular teeth width (−1.5) Mean SDS: maxillary teeth width (−1.4) Small bones and muscles Reduction of thyroid, heart, uterus, and spleen sizes corrected for body surface Ovary and prostate sizes corrected for body surface similar to normal controls Increase of pancreas, liver and kidney sizes corrected for body surface Marked anterior pituitary hypoplasia Ocular axial length of adults corresponds to 96% of the normal controls Reduced anterior chamber depth, vitreous depth Reduced central corneal thickness Increased spherical equivalent and corneal curvature Intraocular pressure and lens thickness similar to controls Reduction of vascular retinal branching points Increase of optic disc Pharyngeal airway of adults similar to normal controls

SDS: standard deviation scores which normal range from −1 to + 1

**Table 2:** Body functions in the Itabaianinha syndrome

Increased energy intake Increased GLP-1 secretion in response to a mixed meal Reduced postprandial ghrelin and hunger attenuation in response to a mixed meal Reduced FGF21 and β-Klotho levels response to a mixed meal. Central and visceral obesity, with fat-free mass reduced Higher locomotor activity Reduced sweating capacity Decreased fat free mass Increased percent body fat Truncal and visceral obesity Increased insulin sensitivity Increased adiponectin Reduced β-cell function No history of neonatal hypoglycemia Increased total and LDL cholesterol Increased C-reactive protein Increased systolic blood pressure in adulthood, and arterial hypertension in the elderly Lack of premature atherosclerosis More prevalent nonalcoholic fatty liver disease, without progress to advanced hepatitis Volumetric bone mineral density, corrected by the size of the bone, similar to controls Higher frequency of genu valgum Better muscle strength parameters adjusted for weight and fat free mass Greater peripheral resistance to fatigue than controls Satisfactory walking and postural balance Normal levels of 25 hydroxy vitamin D and phosphor-calcium homeostasis No spontaneous fractures Less vertebral fractures in elderly Normal visual acuity Normal neural and vascular retina Higher prevalence of dizziness and mild high-tones sensorineural hearing loss Higher prevalence of moderate peripheral vestibular impairment Higher prevalence of the abnormal vestibular-ocular reflex Normal daily immune function Macrophages less prone to *Leishmania amazonensis* infection Similar production of anti-SARS-CoV-2 antibodies, with lower frequency of confirmed cases than in controls Shorter sleep time and more fragmented sleep Normal quality of life No microphallus No history of neonatal hypoglycemia Delayed puberty Anticipated beginning of climacteric Age at menopause similar to control group Preserved fertility Apparently, no breast, colon, and prostate cancers Susceptibility to skin cancer Normal longevity

## BODY SIZE MEASUREMENTS IN ITABAIANINHA SYNDROME

The data in Table 1 show an uneven reduction in bone, as expressed in standard deviation scores (SDSs) and nonbone measures, corrected by body surface. It adds very recent data about the dental arches (29) and the mesiodistal measurement of the teeth (30) to several previously published papers (31–40). The pattern of cephalometric measures explains the doll facies and their high-pitched voice. The reduction in teeth width is of lesser magnitude than height and cephalometric measurements, with the latter two measurements reflecting postnatal growth of bone tissue. The less marked reduction in the size of the teeth coupled to a greater reduction of most jaw dimensions can have deleterious consequences, such as crowding, malocclusion, and periodontal disease (41), but it can have benefits, providing a masticatory advantage. Accordingly, tooth growth parallels ocular axial length and head circumference (brain) development (32), other important elements of environmental adaptation and survival capacity. The growth of the eyes and of the brain seems to be minimally affected by GHD. While the mean stature of affected individuals was 78% of that in the controls, their ocular axial length was 96%, and their head circumference was 92% of the normal local controls. Indeed, ocular axial length reaches its final dimension at approximately 13 years of age (42) before the maximal activation of the somatotrophic axis, while the brain has 83.6% of its growth completed within the first year of life with essentially full growth achieved during the first 3 years of life (43). Tooth growth seems mostly a prenatal process that is partially independent from stature. Therefore, teeth, eye, and brain growth may involve different patterns of temporal regulation than whole-body growth, suggesting other regulatory mechanisms in addition to the somatotropic axis. On the other hand, some organs show size reduction (corrected for body surface): thyroid, heart, uterus, and spleen. Conversely, ovary and prostate sizes were similar to controls (40).

## BODY FUNCTIONS IN ITABAIANINHA SYNDROME

### Skin functions

It is intuitive that the skin, being the covering of the body, is significantly influenced by the somatotrophic axis that controls body size. Skin has many functions, some protective (against microorganisms, dehydration, ultraviolet light, and mechanical damage) and others homeostatic (sweating and production of vitamin D). A mutual influence exists between the skin, growth and the somatotrophic axis, as skin produces IGF1 and vitamin D, and GH and IGF1 exert several actions on the skin (44,45). These untreated IGHD subjects exhibited a reduction in sweating but had normal vitamin D levels and phosphorus-calcium homeostasis (46). In addition, their skin appeared prematurely wrinkled and remained susceptible to cancer (47), as detailed later in this article.

### Muscle function and balance

Although these IGHD individuals had small bones and muscles, their volumetric bone mineral density, corrected for bone size, was normal (48). Additionally, they had better muscle strength parameters (adjusted for weight and fat-free mass) and greater peripheral resistance to fatigue than controls (49). Not surprisingly, there were no reports of spontaneous fractures in this cohort, and the prevalence of vertebral fracture was reduced in older IGHD individuals compared to age-matched controls (50). They presented satisfactory walking and postural balance with no increased risk of falling (51), although they had moderate peripheral vestibular impairment (52) without clinical consequences, as they were quite active in agriculture, horseback riding, and sports.

### Quality of life, reproduction, sleep, and sensory perception

IGHD individuals exhibit normal quality of life (27), despite shorter and more fragmented sleep (53). The external and internal genitalia are essentially normal, which guarantees sexual life with a person of normal stature, with preserved reproductive capacity (20,24,25,54). The organs of sense present a generally very satisfactory performance (little, if any, vision impairment), with mild changes in cochlear function (mild high-tone sensorineural hearing loss) (55) and labyrinth function (moderate peripheral vestibular impairment) (52). These minor problems do not disturb their normal quality of life.

### Body composition, cardio-metabolism, vascular, immune, and cancer data

The changes in body composition include decreased fat-free mass and increased percent body fat (56,57). IGHD subjects eat proportionally more but healthier food than local controls matched for age and gender. In fact, their estimated energy intake corrected by body weight is higher than controls. In addition, they consume, in percentage, more proteins, less carbohydrates, and equal amounts of lipids (58). They show increased areas under the curves of GLP-1 and ghrelin and hunger attenuation in response to a mixed meal (59). They also exhibit reduced FGF21 and β-Klotho levels. These FGF21 and β-Klotho levels may not have been significantly influenced by the test meal but rather reflected their spontaneous morning secretion. This suggests that lower FGF21 and β-Klotho secretion is compatible with healthy status and longevity (60). Together, these “enteroendocrine” connections may result in a favourable outcome in terms of environmental adaptation, ensuring adequate food intake, and may confer metabolic and vascular benefits (59,60).

Despite visceral adiposity (61), these IGHD subjects have increased insulin sensitivity (62), accompanied by high serum adiponectin (63). Insulin sensitivity may contribute to normal longevity (28) but does not prevent the development of diabetes, which is present in 15% of adult IGHD subjects when assessed by OGTT (64), likely due to reduced β-cell function (62). Diabetes has also been reported in patients from the Israeli cohort with Laron dwarfism due to GH insensitivity caused by mutations in the GH receptor gene (65), while there was no self-reported diagnosis of diabetes in the Ecuadorian cohort with the same genetic defect (66). Metabolic fatty liver disease is more prevalent in IGHD adults than in local controls, without progression to advanced forms of hepatitis (67). These IGHD subjects had high serum total and LDL cholesterol levels (57,68). They also exhibited higher circulating C-reactive protein, an increase in systolic blood pressure in adults, and arterial hypertension in older age, without evidence of cardiac hypertrophy or an increase in carotid intima media thickness (57) or coronary (69,70) and abdominal aortic atherosclerosis (50). Cerebral vasoreactivity, a surrogate marker of cerebrovascular disease, was not impaired in these subjects, and IGHD did not affect quantitative measures of the vascular and neural retina (71). Therefore, retinal development, such as in the teeth, eye, and brain, may involve different patterns of regulation than whole-body growth, suggesting other regulatory mechanisms in addition to the somatotrophic axis. All these systems are extremely important for environmental adaptation and are responsible for hierarchical functions.

Immune function is also very important for environmental adaptation and survival capacity. Accordingly, we did not observe significant immune deficits in this cohort, especially for the most prevalent pathogens in the region. We observed no difference between IGHD and controls regarding a history of infectious diseases, baseline serology, and in the response to hepatitis B, tetanus, and bacillus Calmette-Guérin vaccinations or in the positivity to PPD, streptokinase or candidin skin tests (72). These IGHD subjects have a higher prevalence of periodontal disease than local controls, probably caused by their dental crowding (41). The apparently normal immune function suggests that many immune cells use extrapituitary circuits (local GH/IGFs), independent from the somatotrophic axis. We also found that macrophages from IGHD subjects are less prone to *Leishmania* *amazonensis* infection than GH-sufficient controls (73) and that they appear to cope better with SARS-CoV-2 infection than controls (74). Resistance to *Leishamnia* infection may be one of the reasons for the spread of this mutation in the Itabaianinha region.

In the entire IGHD Itabaianinha cohort, during 28 years of medical care, our team did not diagnose any cases of breast, colon, or prostate cancer (20,24,25). The absence of these common neoplasms suggests that GH and IGF1 deficiency protects against DNA damage and favours apoptosis of damaged cells, thus reducing the risk of cancer. Thus far, we have found one IGHD subject with a skin tag, which was found to be a fibroepithelial polyp by pathological examination, and seven epidermoid skin cancers, one lethal, indicating a vulnerability of their skin to tumour development (47). Additionally, a 25-year-old woman who had intermittently received GH replacement therapy from age 11 to 18 developed an ependymoma extending from the fourth ventricle to the end of the thoracic spine. She underwent three surgical procedures without obvious evidence of tumour recurrence during the 10-year follow-up.

### Healthspan and lifespan

Although it is intuitive that geriatric medicine seeks to extend lifespan, in the last three decades, its main strategy has been the compression of morbidity. This strategy delays the age of onset of chronic disease and disability rather than increasing survival, limiting morbidity to a shorter period and closer to the end of life, thus reducing the total amount of disease and disability. More recently, the theory of morbidity compression has evolved to promote the concept of healthspan, that is, the period of life free from major chronic clinical diseases and disabilities. To achieve optimal longevity (long life, but primarily well-being), the duration of life without significant comorbidities (healthspan) must be significantly extended (75).

IGHD individuals from Itabaianinha are very active throughout their lives and generally have a healthy old age, with an extended healthspan and a lifespan comparable to that of their relatives without GHD (28). Some are centenarians, and many of those who die at an advanced age die from external causes, such as accidents or preventable conditions (25). Therefore, these individuals constitute a model of optimal longevity in light of modern geriatrics (long life, but mainly with well-being). These data are complementary to the extensive experimentation led by Dr Andrej Bartke of Southern Illinois University School of Medicine, Springfield, which showed that IGHD mice due to GHRH or GHRH receptor mutations and mice with GH resistance live longer than their normal siblings with an extended healthspan (25).

### MicroRNAs signatures

MicroRNAs (miRNAs) are important regulators of metabolism and healthy ageing (76). MicroRNAs are short noncoding RNA segments that can induce target mRNA cleavage and translational repression and play a central role in the posttranscriptional regulation of cell function (77). They can be measured in the systemic circulation, where they can act as endocrine hormones regulating various physiological processes. Circulating miRNAs can also target genes in cells of different tissues and organs. The signature of circulating miRNA can potentially serve as a noninvasive diagnosis of chronic diseases, such as cancer, diabetes, and cardiovascular disease (78,79).

We found a significant regulation of age-related miRNAs in Itabaianinha IGHD subjects (80). These miRNAs have an important overlap with serum-regulated miRNAs in GH-deficient mice, which have a remarkable extension of healthspan and lifespan (81). Of note, the target genes predicted for serum-regulated miRNAs in IGHD subjects contribute to insulin-, inflammation-, and ageing-related pathways, such as the mTOR and FoxO pathways. The main upregulated age-related miRNAs, miR-100-5p, miR-195-5p, miR-181b-5p and miR-30e-5p, have been found to regulate the in vitro expression of the age-related genes mTOR, AKT, NFκB and IRS1. Therefore, normal longevity is mirrored by a favourable miRNA signature.

### Roles of the components of the somatotrophic system in body size and body functions

Table 3 shows in simplified form the roles of the components of the somatotrophic system in body size and body functions. The somatotropic axis is crucial for body size and composition and skin and is important for some body functions, such as metabolism, voice production and auditive and vestibular functions. On the other hand, extrapituitary circuits are crucial for the growth of some organs, such as teeth, eyes and the brain.

**Table 3:** Simplified Scheme of the roles of components of the somatotrophic system

SOMATOTROPIC AXIS PITUITARY GH & CIRCULATING IGF1	EXTRA-PITUITARY CIRCUITS INSULIN, IGF2, LOCAL GH/IGFS & GROWTH FACTORS
Crucial for body size	Crucial for hierarchy functions
Stature	Immune
Body Composition	Brain Function
Skin	Reproduction
Thyroid, Heart, Spleen, Uterus Size	Eyesight
**Important for functions**	**Important for sizes**
Metabolism	Fetus and Birth Size
Voice Production	Brain
Auditive Function	Eye
Vestibular Function	Teeth

In conclusion, Sergipe has contributed to the study of GH excess (Souza Leite) and GH deficiency (with the description of Itabaianinha syndrome). This last line of research, lasting almost thirty years, has sought to establish the role of the components of the somatotrophic system in body size and body functions. The balance of conditions associated with this severe and congenital IGHD shows that the benefits outweigh the harms. Our hypothesis is that having very little exposure to GH throughout life may be more advantageous than having normal GH secretion followed by a decline caused by an acquired pituitary insult.
